# A cluster-randomized study to evaluate the effectiveness and cost-effectiveness of the Assessment of Burden of Chronic Conditions (ABCC) tool in South Tyrolean primary care for patients with COPD, asthma, type 2 diabetes, and heart failure: the ABCC South Tyrol study

**DOI:** 10.1186/s13063-024-08041-9

**Published:** 2024-03-20

**Authors:** Christian J. Wiedermann, Pasqualina Marino, Angelika Mahlknecht, Verena Barbieri, Giuliano Piccoliori, Adolf Engl, Annerika H. M. Gidding-Slok

**Affiliations:** 1 Institute of General Practice and Public Health, Claudiana—College of Health Professions, Bolzano (BZ), Italy; 2 Department of Family Medicine, Care and Public Health Research Institute (CAPHRI), Maastricht University, Maastricht, The Netherlands

**Keywords:** Chronic diseases, Primary care, Patient-reported outcome measures, Assessment of Burden of Chronic Conditions tool, Quality of care, Quality of life, Self-management, Health care utilization, Cost-effectiveness, Health technology assessment

## Abstract

**Background:**

Chronic diseases, such as chronic obstructive pulmonary disease (COPD), asthma, type 2 diabetes, and heart failure, often coexist and contribute to a significant burden on individuals and health systems. The Assessment of Burden of Chronic Conditions (ABCC) tool, already in routine clinical use in the Netherlands, aims to comprehensively assess and visualize disease burden, stimulate self-management, and encourage shared decision-making. This study aims to validate the German and Italian versions of the ABCC tool and evaluate its effectiveness and cost-effectiveness in the South Tyrolean Primary Care setting.

**Methods:**

This is a cluster-randomized study involving approximately 400 patients with COPD, asthma, type 2 diabetes, and heart failure who received care from the South Tyrolean General Practices. Initially, the ABCC tool will be translated into German and Italian and validated. Subsequently, half of the participants will use the validated ABCC tool for patient-reported outcome measurement assessments, while the other half will receive usual care. The primary outcome measure is the change in the patients’ perception of the quality of care after 18 months. The secondary outcomes included changes in quality of life, self-management behavior, and healthcare utilization. The missing data will be managed using multiple imputations. Additionally, a cost-effectiveness analysis that considers the direct medical costs reimbursed by the National Health Service will be conducted.

**Discussion:**

This study provides insights into the application, validation, and efficacy of the ABCC tool in the South Tyrolean healthcare context. The tool’s potential to enhance person-centered care, improve the quality of life, and possibly reduce healthcare costs could greatly contribute to sustainable healthcare. The challenges of implementation, such as software integration and the use of an EU data platform, will provide lessons for future international patient care data management.

**Trial registration:**

ISRCTN registry, ISRCTN13531607. Registered on August 23, 2023.

**Supplementary Information:**

The online version contains supplementary material available at 10.1186/s13063-024-08041-9.

## Administrative information

Note: the numbers in curly brackets in this protocol refer to SPIRIT checklist item numbers. The order of the items has been modified to group similar items (see http://www.equator-network.org/reporting-guidelines/ spirit-2013-statement-defining-standard-protocol-itemsfor- clinical-trials/).

**Table:** 

Title {1}	A Cluster-Randomized Study to Evaluate the Effectiveness and Cost-Effectiveness of the Assessment of Burden of Chronic Conditions (ABCC) Tool in South Tyrolean Primary Care for Patients with COPD, Asthma, Type 2 Diabetes, and Heart Failure: The ABCC South Tyrol Study.
Trial registration {2a and 2b}	ISRCTN registry, ISRCTN13531607. Registered on 23 August 2023.
Protocol version {3}	08.07.2023 version 2
Author details {5a}	Christian J. Wiedermann, Pasqualina Marino, Angelika Mahlknecht, Verena Barbieri, Giuliano Piccoliori, Adolf Engl Institute of General Practice and Public Health, Claudiana – College of Health Professions, Bolzano (BZ), Italy Annerika H.M. Gidding-Slok Department of Family Medicine, Care and Public Health Research Institute (CAPHRI), Maastricht University, Maastricht, The Netherlands
Name and contact information for the trial sponsor {5b}	Institute of General Practice and Public Health Claudiana – College of Health Professions Lorenz Böhler Street 13 39100 Bolzano (BZ), Italy Email info@am-mg.claudiana.bz.it Tel. + 39 0471 067 392
Role of sponsor {5c}	The study is sponsored by the Claudiana – College of Health Professions in Bolzano, Italy. The sponsoring institution has no particular influence on the study design; the collection, management, analysis, and interpretation of data; the writing of the report; or the decision to submit the report for publication. Ultimate authority over all these activities rests solely with the Institute of General Practice and Public Health.

## Introduction

### Background and rationale {6a}

Chronic diseases pose a significant burden on healthcare systems globally [1]. This situation is similar to that in South Tyrol, Italy [2]. The complexity of chronic disease management often results in inefficient care, patient dissatisfaction, and increased healthcare costs [3]. Therefore, there is an urgent need for an effective and integrated care model for managing chronic diseases [4]. This has led to the emergence of the Chronic Care Model (CCM), which has been proven to provide better health outcomes for patients and improve efficiency in healthcare systems [5].

To enhance the effectiveness of CCM, the Assessment of Burden of Chronic Conditions (ABCC) tool has been developed [6]. This tool has been successfully implemented in the Netherlands, demonstrating its potential benefits in chronic disease management [7]. It is currently available through various providers, including hospital information systems and personal health environments [8]. However, its implementation in other regions, such as South Tyrol, has yet to be explored.

The ABCC tool promises to improve chronic care management by capturing and integrating patients’ comprehensive health information into a digital platform. This comprehensive approach may facilitate a more individualized and person-centered care plan, leading to improved health outcomes [7]. Furthermore, the integration of ABCC findings into an electronic health record (EHR) system ensures that information is secure, accurate, and accessible, thereby enhancing the delivery of care [9, 10].

Despite its potential benefits, the implementation of the ABCC tool in the CCM in South Tyrol presents several challenges. These include the adaptation of the tool to German and Italian languages, digitalization of the tool, validation of the tool on patients, study of its efficacy and cost-effectiveness in general practice, and integration into the electronic health record (EHR).

Through this project, we aim to contribute to the body of knowledge on integrated chronic care models and provide valuable insights into the use of digital tools in chronic disease management. Ultimately, the successful implementation of the ABCC tool could represent a significant step towards improving the quality of care for patients with chronic diseases in South Tyrol and beyond.

## Objectives {7}

The primary objective of this project is to evaluate the effectiveness of the Dutch ABCC tool adapted for the South Tyrol’s general practice settings. This evaluation will focus on determining the impact of the culturally translated and regionally validated ABCC instrument on patients with chronic diseases in South Tyrol. The key research question is, ‘‘How does the adapted ABCC tool affect perceived quality of care and the need for further health services in these patients, compared with usual care, after 18 months?” Should this adapted tool prove effective, our aim is to implement it in primary care and integrate its findings into electronic health records systems.

## Trial design {8}

This study is designed to explore the effectiveness and cost-effectiveness of the ABCC tool in primary care settings in Southern Tyrol. It will follow a pragmatic, cluster-randomized design, with two distinct groups: an intervention group that will utilize the ABCC tool and a control group that will adhere to the regular care regimen. Since the ABCC tool has not yet been integrated into South Tyrolean healthcare, theoretically, all general practitioners could participate in both intervention and control groups. Following the PRECIS-2 framework, details of the trial components are provided as a supplementary material (Additional file 1) to the manuscript, including setting, participant recruitment, intervention delivery, follow-up, and outcomes as defined by Loudon et al. [11].

To mitigate potential contamination bias—whereby patients in the control group could inadvertently receive the intervention (or vice versa) due to overlapping care by health care providers—the randomization of the intervention will be performed at the level of general practices [12]. In South Tyrol, general practitioners (GPs) work either independently in their private practices or in group practices. However, even within group practices, GPs work independently, each with their own designated patient lists provided by the public health service.

Outcomes will be monitored and evaluated 18 months after implementing the ABCC tool in the intervention group, and these results will be compared with those of the control group receiving usual care. This study aims to assess the comparative effectiveness and cost-effectiveness of the ABCC tool compared to standard care for patients with chronic diseases in South Tyrol’s general practice setting.

## Methods: participants, interventions and outcomes

### Study setting {9}

The Health Authority of South Tyrol, a region with a mix of Italian and Austrian cultures, is essentially part of the Italian National Health Service (NHS). In this region, general practices are predominantly independent entities, often run by single practitioners or small groups of GPs who work independently but may share office space. These practices are not typically housed within hospitals or larger medical centers, maintaining a focus on community-based care. Patients are assigned to GPs by the public health service, and GPs are paid on a per capita basis for the number of patients assigned. This study will be conducted in primary care facilities in South Tyrol. Data will be collected from patients in the general practice in this region. Researchers will enroll in general practices with participation from healthcare providers, such as general practitioners and practice nurses. Practice randomization will be stratified. Subsequently, healthcare providers will recruit the patients. This is because both the provider and the patient will either use the ABCC tool (intervention group) or receive standard care (control group). The study outcomes are assessed at the practice and patient levels.

### Eligibility criteria {10}

Eligibility criteria include patients with a diagnosis of chronic obstructive pulmonary disease (COPD), asthma, type 2 diabetes mellitus (T2DM), and/or heart failure, who are 18 years of age or older, and who understand and read German or Italian. Exclusion criteria include patients with asthma or COPD who had taken prednisone for an exacerbation within 6 weeks prior to the start of the study and patients with T2DM or heart failure who had been hospitalized within 6 weeks prior to the start of the study.

Eligibility criteria for participants will be validated through manual chart review, owing to the variability in software programs used across general practices and the lack of a unified EHR system in the region.

### Who will take informed consent? {26a}

Informed consent will be obtained by either the General Practitioner (GP) investigator or the General Practice Assistant involved in the study. These individuals will be comprehensively trained on how to obtain their informed consent. This training emphasizes the importance of transparency and open communication during the consent process, the need to respect participant autonomy, and the need to ensure that participants fully understand the study’s implications.

The consent process will involve potential participants who have received an informed consent form approved by the Ethics Committee. This form contains detailed information about the trial, including its purpose, duration, procedures, and associated risks and benefits. Participants will be given the opportunity to read this document alone or with the GP investigator or a General Practice Assistant.

They will be able to ask any questions they may have about the trial and the GP investigator or General Practice Assistant will provide comprehensive answers to these queries. Once the participants are comfortable and have confirmed their understanding of the study, they will be asked to provide written consent to participate. This procedure ensures that the consent obtained is informed, voluntary, and in accordance with the guidelines.

### Additional consent provisions for collection and use of participant data and biological specimens {26b}

This study requires the collection and use of the participant data. As part of the informed consent process, participants will be informed about the data collection procedures, the type of data that will be collected, how they will be used, and how their privacy will be protected.

Participants will be asked for their consent by the research team to use their anonymized data for the purposes of this study. They will also be informed that their participation is voluntary and that they have the right to withdraw their consent at any stage of the study without any negative implications for their care.

The consent form contains provisions for any potential ancillary study that may arise from the primary study. If such ancillary studies involve additional data collection or usage, separate consent will be sought from participants at that time. This approach ensures that the participants were fully aware of how their data would be used, providing them with control over their personal information.

## Interventions

### Explanation for the choice of comparators {6b}

The comparator in this study is the current standard of care for managing COPD, asthma, T2DM, and heart failure. Current standard of care follows integrated care protocols developed by interprofessional groups that include disease diagnosis, monitoring, and treatment pathways. However, these protocols do not include the use of patient-reported outcome tools, which the study intervention seeks to integrate for a more comprehensive assessment of patient health outcomes. Comparing the ABCC tool with the current standard of care allows us to evaluate its added value and efficacy in a real-world setting. If the ABCC tool proves to be superior or equivalent to the current standard of care, it would provide a compelling argument for its integration into routine clinical practice. The use of the current standard of care as a comparator ensured that all study participants received a level of care aligned with the best current practices. This approach respects ethical considerations by ensuring that no participant is deprived of a known effective treatment. By comparing outcomes, resource utilization, and costs between the intervention and the current standard of care, we can gain insights into the potential benefits and impacts of the wide-scale implementation of the ABCC tool.

### Intervention description {11a}

This study involves the use of the ABCC tool. This tool is a patient-reported outcome measure (PROM) designed to assist in individualized medical decision-making within a complex and changing healthcare environment. The ABCC tool is an evolution of the Assessment of Burden of COPD (ABC) tool developed in 2014 [13] and has been expanded to cover various chronic conditions beyond COPD, including asthma, type 2 diabetes mellitus (T2DM), heart failure [6], and post-COVID syndrome [8].

The ABCC tool integrates a generic module with disease-specific modules to facilitate a comprehensive approach to patient care. Table 1 lists the four key components, their explanations, and illustrative descriptions to provide insight into how each component supports patient-centered care during the consultation.

**Table 1:** Components of the ABCC Tool in consultation sessions

**Component**	**Explanation**	**Illustrative description**
Assessment	Utilizes a short scale to assess the burden of disease due to one or more chronic conditions	Questionnaire
Visualization (balloon diagram)	Self-assessment results are visualized using balloons of varying colors and heights to represent scores for items or domains. Green for good scores, red for areas of difficulty, and gray for previous scores to indicate change	Imagine a balloon for each health domain: green balloons rise high for areas of well-being, while red balloons hover low for challenges
Shared decision-making discussion	A discussion between the patient and healthcare provider, supported by treatment recommendations integrated into the tool	Guided conversation on treatment options based on assessment results
Agreement with personalized care targets	Setting individualized care goals based on the patient’s self-assessment and shared decision-making discussions	A section within the tool where patients and providers document agreed-upon health goals

The ABCC tool uses the ABCC scale, which includes general, disease-specific, and lifestyle questions to assess a patient’s disease burden. Access to participant responses and balloon visualizations is provided through an online portal, accessible on both tablet PCs for patient surveys and GP office desktop PCs, with the option for printing to facilitate in-depth discussions during consultations.

#### Intervention translation and validation

The scale has been validated in Dutch and found to correlate significantly with the Saint George Respiratory Questionnaire (SGRQ), the Standardised Asthma Quality of Life Questionnaire (AQLQ-S), and the Audit of Diabetes Dependent Quality of Life Questionnaire (ADDQoL19) [6]. It has also shown high internal consistency and good test–retest reliability [9].

The protocol includes steps to translate and validate the Dutch ABCC tool into German and Italian versions. The creators of the ABCC tool, developed with funding from ZonMw, a Dutch funder, granted permission for free use in both research and routine patient care.

A Dutch scientific service provider will assist with the cross-cultural adaptation of the ABCC questionnaire because of the team’s limited Dutch proficiency. A forward–backward translation procedure will be applied, involving independent translations into Italian or German and back-translations into Dutch. Post-translation cognitive interviews with a small group of chronically ill individuals will assess the content validity from the patient’s perspective.

To validate the translated ABCC tools, we will employ the “COPD Population Screener™” (COPD-PS™) and the “Asthma Control Test™” (ACT™) [14], both available in Germany and Italy [15, 16], to evaluate convergent validity. For T2DM patients, we will use the “European Quality of Life 5 Dimensions 5 Level” (EQ-5D-5L) questionnaire, while the “Kansas City Cardiomyopathy Questionnaire” (KCCQ) will be used for heart failure patients. In both cases, validated German and Italian versions are available [17–20]. Following the use of disease-specific measures for each condition, the approach to validating the convergent validity of the ABCC tool will be to analyze correlations between ABCC tool scores and these established non-ABCC measures.

We aim to include 30 persons per disease group in line with the initial ABCC scale validation in the validation study. Eligible patients are those diagnosed with COPD, asthma, T2DM, and/or heart failure who are 18 years or older and comprehend and read German or Italian. As the ABCC tool is a monitoring tool and not a diagnostic tool, both new and existing patients are suitable. To ensure participant stability at baseline, those who had taken prednisone for asthma or COPD exacerbation or were hospitalized for T2DM or heart failure, within 6 weeks prior to the start of the study will be excluded.

### Criteria for discontinuing or modifying allocated interventions {11b}

Given that the ABCC tool is used and then the participant is followed up with measurements for up to 1 year, the criteria for discontinuation of the intervention itself are as follows: (i) participants have the right to withdraw their consent at any time, for any reason. If a participant wishes to discontinue the use of the ABCC tool after it has been administered but before the follow-up period, their request should be honored. (ii) If a participant experiences significant emotional distress during the initial administration of the ABCC tool that cannot be adequately addressed, intervention discontinuation is warranted. (iii) If a participant’s health status changes significantly immediately after using the ABCC tool such that the tool is no longer appropriate or relevant, discontinuation of the intervention might be necessary. (iv) If a technical error occurred during the initial administration of the ABCC tool that rendered the results invalid or unusable and it is not feasible to re-administer the tool, discontinuation of the intervention might be the best course of action.

In cases where discontinuation of the ABCC tool intervention is being considered, the decision will be made through a collaborative process involving the participant and their GP. The coordinating center is available to provide guidance and support in this decision-making process, ensuring compliance with study protocols and ethical standards.

### Strategies to improve adherence to interventions {11c}

Adherence to the ABCC intervention and follow-up measurements will be improved and monitored by (i) ensuring that participants understand the importance of the ABCC tool and the role played by their participation in the research. This includes explaining the purpose and benefits of the tool, the potential impact of the research on healthcare, and how their contributions can make a difference. (ii) Regular contact with the participants should improve adherence. Reminders about upcoming follow-up measurements will be sent, checking in with participants to see how they are doing and answering any questions they may have. (iii) The Dutch ABCC tool is easy to use and visually appealing. The tool will be used in the South Tyrol study with an identical design to encourage its use. (iv) The tool is easily accessible to all participants regardless of their technological expertise or resources. This will involve providing options for in-person, phone, or online follow-up if possible. (v) Monitoring adherence is digitally tracked and follow-up calls are made. All strategies will comply with ethical guidelines and data protection regulations, and respect participant confidentiality and autonomy.

### Relevant concomitant care permitted or prohibited during the trial {11d}

All concomitant care and interventions will be reported in this study. The primary concern is the safety and well-being of participants. If treatment intervention or care is necessary for the participant’s health, it will not be prohibited, even if it may potentially impact the trial results. The trial management team will regularly monitor and review concomitant care and interventions. This will help identify any potential interactions with ABCC intervention. Participants, caregivers, and the healthcare team will be educated about concomitant care and interventions, which will help ensure compliance and protect the participants’ health. All instances of concomitant care and intervention will be thoroughly documented.

### Outcomes {12}

#### Primary outcome

The primary objective of this study is to assess changes in perceived quality of care over an 18-month period using the Patient Assessment of Chronic Illness Care (PACIC) [20, 21] as the comparative measure. This assessment will be administered at baseline and then at specified follow-up points to determine changes from usual care in the cohort of participants.

#### Secondary outcomes

Secondary objectives encompass several areas:

1. Assessing the shift in perceived quality of care, employing the PACIC, against regular care after 18 months, individually for each chronic condition.
2. We evaluate variations in perceived quality of care, gauged by the PACIC, against regular care at 6- and 12-month intervals for both the entire participant group and each chronic condition individually.
3. Evaluate changes in universal health-related quality of life, as gauged by the EQ-5D-5L [21, 22], in comparison with standard care at 6-, 12-, and 18-month intervals for the entire participant group and for each chronic condition separately.
4. Scrutinizing changes in participant activation, as measured by the Patient Activation Measure (PAM) [23, 24], against standard care at 6-, 12-, and 18-month intervals for both the entire participant group and for each chronic condition.
5. Conducting practice-level analyses to evaluate the overall impact of the ABCC tool on healthcare delivery and outcomes. This includes assessing changes in practice-wide metrics for average improvements in PACIC scores, EQ-5D-5L outcomes, and PAM levels, comparing the aggregated results of the intervention group practices against those of the control group practices.

In addition, we will assess the cost-effectiveness of the ABCC tool compared with usual care after an 18-month period. This analysis focuses on the direct medical costs reimbursed by the National Health Service (NHS). The economic resources claimed will be obtained from the data of general practitioners, the health administration of the province, and the South Tyrolean ambulance service. Only costs and benefits from the healthcare perspective are considered and will include the following:

- Pharmaceutical treatments for chronic disease(s): The cost of purchasing branded medicines will be determined from national price lists, with public prices used after subtracting the discounts prescribed by the Agenzia Italiana del Farmaco (AIFA). For generic medicines, we used prices from the AIFA transparency list, which lists medicines reimbursed by the NHS, based on the reference price system.
- Hospitalization for chronic disease(s): Costs are determined using the latest national DRG tariff system of the Italian Ministry of Health. This system quantifies resource use and provides an estimate of the cost per acute event from an NHS perspective.
- Outpatient specialist visits and examinations for chronic disease(s): Costs will be based on the latest available “National Tariff Nomenclator.”
- General Practitioner (GP) visits for chronic disease(s): Costs are taken from Dal Negro et al. [25] and extrapolated to current prices according to the ISTAT consumer price index.

### Participant timeline {13}

Participants’ timeline for this study is as follows (Table 2):

**Table 2:** SPIRIT schedule of enrolment, interventions, and assessments of the ABCC South Tyrol study

	**STUDY PERIOD**						
	**Enrolment (Pre-allocation)**	**Allocation**	**Post-allocation (Months)**				**Close-out**
**TIMEPOINT**	***-1***	**0**	***0***	***6***	***12***	***18***	***18***
**ENROLMENT:**							
**Practice agreement**	X						
**Practice allocation**	X						
**Patient eligibility screen**	X						
**Patient informed consent**	X						
**INTERVENTIONS:**							
**Regular Care**			X				
**ABCC Care**			X				
**ASSESSMENTS:**							
**Chronic Condition**		X					
**PACIC**		X		X	X	X	X
**EQ-5D-5L**		X		X	X	X	X
**PAM**		X		X	X	X	X
**Direct Medical Costs**							X

*ABCC* Assessment of Burden of Chronic Conditions, *EQ-5D-5L* European Quality of Life 5 Dimensions 5 Level, *PACIC* Patient Assessment of Chronic Illness Care, *PAM* Patient Activation Measure

#### Enrollment and baseline assessment

The recruitment and enrollment process will begin once the general practices are randomized to either the intervention or control group. Baseline assessments will be conducted for all enrolled participants. This assessment will include measurement of perceived quality of care using the PACIC, evaluation of health-related quality of life using the EQ-5D-5L, and assessment of patient activation using the PAM.

#### Intervention period

The intervention period will start immediately after the baseline assessment for participants assigned to the intervention group. These will be introduced into the ABCC tool and will provide guidance on how to use it. The control group continued to receive usual care.

#### Follow-up assessments and visits

All participants (both intervention and control groups) will undergo follow-up assessments at 6, 12, and 18 months post-baseline. These assessments measure the following.

1. Perceived quality of care using the PACIC.
2. Health-related quality of life using the EQ-5D-5L.
3. Patient activation using the PAM.

At each follow-up visit, any changes or updates to participants’ health conditions or care regimens were noted. Follow-up visits will also serve as opportunities for researchers to address any questions or concerns that the participants may have and ensure that the participants in the intervention group used the ABCC tool correctly.

#### End of study

The study will conclude after the 18-month follow-up assessment, at which point the primary and secondary outcomes will be evaluated. The primary outcome will be the perceived quality of care as measured by PACIC for the entire participant group and for each chronic condition separately. Changes in quality of life (EQ-5D-5L) and patient activation (PAM) at different time points led to secondary outcomes.

The expected duration of the entire study, including the enrollment, intervention, evaluation, and close-out phases, is expected to be two and a half years. This timeframe includes the pre-allocation preparation phase, the 18-month period for implementation of interventions and conduct of assessments, and additional months for initial recruitment and final analysis.

### Sample size {14}

The sample size estimation was guided by analogous assumptions from a concurrent study conducted in the Netherlands, thereby offering a relevant frame of reference for our calculations. The study’s primary outcome will be determined through the Patient Assessment of Chronic Illness Care (PACIC) measure. Given the lack of substantial data on the minimum significant difference and standard deviation for the PACIC in this specific population, an estimated medium effect size of 0.51 is derived from the work of Slok et al. [26]. This is based on an observed mean difference of 0.49 between the intervention and control groups, and a pooled within-group standard deviation of 0.96 from the same study.

For the current study, using an independent samples *t*-test, a significance level of 0.05 for two-sided testing, a power of 90%, and an even split between intervention and control groups, it is estimated that each group should include 82 patients. However, as patients will be grouped within general practices, this number is adjusted to account for the design effect, which is calculated as 1 + (m − 1) × ICC (intraclass correlation), where an ICC of 0.05 and an average of 10 patients per general practice is assumed [6].

After adjusting for cluster design (multiplication factor of 1.45), variable cluster sizes (division by 0.9), and an anticipated dropout rate of 25% (division by 0.75), it was estimated that each group would require 177 participants. Each group will need to include 18 general practices with an average of 10 patients per general practice. Therefore, 360 participants (180 per group) from 36 general practices (18 per group) will be recruited for this cluster-randomized study.

The aim was to achieve a balanced distribution among the various chronic conditions. However, given that healthcare providers are not explicitly instructed to strive to achieve this balance, this may not be ensured.

### Recruitment {15}

Several strategies will be employed to ensure that the target sample size is reached. The existing relationships between health networks and medical associations in South Tyrol will also be leveraged. The research team will directly contact general practitioners in South Tyrol to invite them to participate in the study through meetings, letters, email, and phone calls. The aim is to provide them with detailed information about the study, address any concerns, and encourage them to enroll their patients. While maintaining ethical considerations, we may provide appropriate incentives to general practitioners for their involvement in the study, including recognizing their contribution to the study, offering educational opportunities related to chronic disease management, and reporting findings that might be beneficial for their practice. Once general practitioners agree to participate, they are effectively “consented” into the study and the research team will maintain regular communication with them to ensure that they have the necessary support to recruit and enroll patients, and address any issues that may arise during the study period.

Patient understanding and consent are crucial for patient enrollment. Thus, we will ensure that potential patients receive clear, easy-to-understand information about the study, its benefits, and what their involvement entails. Patient participants will be given sufficient time to consider their participation and ask questions.

These strategies are dynamically adapted based on the enrollment rate to ensure that the target sample size is achieved within the planned timeline.

## Assignment of interventions: allocation

### Sequence generation {16a}

In this cluster-randomized trial, general practices throughout South Tyrol serve as the unit of randomization. After recruitment, these practices will be assigned to either the intervention or control group through a stratified randomization process. Stratification factors include the patients’ preferred language (Italian or German), the location of the practice (urban or rural), and the demographic characteristics of the general practitioners (sex and age). Sequences will be generated using computer software to pre-generate random assignments. Practices in the intervention group will use the ABCC tool via a provided tablet PC, while those in the control group will continue their usual care without access to the tool.

To maintain the integrity of this randomization process and control for potential confounding, these stratifying variables will be explicitly included in the analytical models.

### Concealment mechanism {16b}

In this study, the allocation sequence was implemented using a centralized, computer-generated randomization process. This process will be managed by a dedicated statistician who will not be involved in participant recruitment or intervention delivery, thereby ensuring concealment of the sequence until interventions are assigned.

Once the general practices have been recruited, demographic and practice characteristics (including the preferred language of patients, urban or rural location, and demographic characteristics of the general practitioners) will be collected and entered into randomization software. The software then generates a random allocation sequence, assigning practices to either the intervention group (use of the ABCC tool) or the control group (usual care without the ABCC tool).

The allocation sequence will be kept confidential and will not be disclosed to the general practice or research team involved in the recruitment and follow-up of participants until the assignment point. This approach ensures that the assignment process is not influenced by any pre-existing knowledge or bias and that allocation concealment is maintained throughout the study.

Once the randomization process is completed, each general practitioner will be informed of their group assignment by the central research team and the necessary instructions or equipment (including the tablet PC for the intervention group) will be provided. This method ensured that the allocation process was transparent and unbiased, thereby maintaining the integrity of the study design.

### Implementation {16c}

Participant enrollment and group allocation will be managed directly by the research team. After generation of the random allocation sequence, practices will be notified of their assignment to either the intervention or control group. Necessary equipment, including a tablet PC loaded with the ABCC tool, along with instructions for use, will be provided to practices in the intervention group to ensure a clear and efficient setup for the conduct of the study.

## Assignment of interventions: blinding

### Who will be blinded {17a}

In this study, blinding of trial participants and care providers (i.e., general practitioners and practice nurses) is not possible because of the nature of the intervention, as they will know whether they are using the ABCC tool or delivering standard care.

However, outcome assessors and data analysts will be blinded to the assignment of the interventions. This is achieved by coding the groups in the dataset in a manner that does not reveal their assignments (using non-descriptive labels, Groups A and B). The individual who assigns these codes maintains a separate, secure record of the code that corresponds to the treatment group.

This record will not be shared with outcome assessors and data analysts. The outcome assessors, who will evaluate the results of the patients’ self-reported outcomes, and the data analysts, who will perform statistical analyses of the collected data, will be blinded to the allocation.

### Procedure for unblinding if needed {17b}

In this study, only the outcome assessors and data analysts are blinded. Because the trial participants and care providers are not blinded, there is no need for unblinding procedures in these groups.

For outcome assessors and data analysts, unblinding may be permissible under specific circumstances such as serious adverse events that require immediate knowledge of the participant’s intervention group to ensure appropriate medical care. Although the need for unblinding may be relatively low due to the nature of the intervention (ABCC tool versus standard care), having an established unblinding procedure in place ensures that any unforeseen circumstances can be managed appropriately without compromising the overall study design. An independent designated person will be identified as responsible for managing the unblinding process, ensuring that it occurs only when the documentation of any unblinding event is justified and maintained. Each designated person evaluates each request for unblinding. If the request is approved, the designated person will access the secure record with the group allocation codes and disclose the participant’s intervention group only to relevant individuals who require information to manage the situation.

## Data collection and management

### Plans for assessment and collection of outcomes {18a}

The assessment and collection of outcomes will be performed by using well-established survey instruments. These include the Patient Assessment of Chronic Illness Care (PACIC) as the primary outcome, that is, perceived quality of care. Secondary outcomes such as health-related quality of life, patient activation, and capability well-being will be assessed using EQ-5D-5L and Patient Activation Measure (PAM), respectively. Data collection will follow a set schedule with checkpoints at 6-, 12-, and 18-month intervals from the start of the intervention to monitor changes in these outcomes over time.

The research team will facilitate survey completion through telephone or email contact, offering participants the option to complete surveys online or on paper. In addition, participants visiting their primary care physician’s office will have the option of using the ABCC tablet to complete the survey during their visit.

### Plans to promote participant retention and complete follow-up {18b}

Retention of participants and ensuring complete follow-up will be achieved by employing various strategies [27]. We will maintain regular communication with the participants, provide them with feedback about the progress of the study, and recognize their contributions to the research. Furthermore, the data collection schedule was designed to minimize participant burden, and we will accommodate participants’ needs wherever possible to encourage continued involvement.

### Data management {19}

A robust data management plan is in place to store, organize, and access the collected data. We will use a secure, centralized data management system for these purposes, and only authorized study personnel will have access to it [28]. In addition, regular data audits will be conducted to identify and correct any data entry errors or inconsistencies, thereby ensuring data integrity.

### Confidentiality {27}

To protect participants’ confidentiality, all personal identifying information is separated from the study data and replaced with unique study identifiers. Access to personal information is limited to the essential personnel. All data will be stored securely in compliance with data protection regulations, and any report or publication will contain only aggregated data, with no possibility of identifying individual participants. After the trial has ended, all personal identifying information will be securely destroyed, and anonymized study data will be preserved for a predefined period as per regulatory guidelines, ensuring ongoing confidentiality.

## Statistical methods

### Statistical methods for primary and secondary outcomes {20a}

The data evaluation process is based on the intention-to-treat principle. Initial analyses will be performed on the entire group of patients with COPD, asthma, type 2 diabetes, and heart failure, which also includes those with multiple conditions. Subsequently, the impact of the ABCC tool is examined separately for patients with COPD, asthma, type 2 diabetes, and heart failure, irrespective of the presence of coexisting conditions.

Considering the clustered design of the study, a three-level multilevel analysis will be conducted: general practice, patient, and measurement. The study will apply linear mixed models to analyze outcomes to account for the grouping of measurements within patients in general practice. Fixed factors will include “treatment group,” “time” (categorical), and “interaction between treatment group and time.”

Given that randomization of patients into the intervention or control groups is not feasible, factors believed or known to influence the perceived quality of care and/or the use of the ABCC tool will be regarded as potential confounders. The intervention or control group assignment hinges on the accessibility of the ABCC tool (i.e., the presence of the ABCC tool tablet PC in general practice). Potential confounders include ethnic background (native/Western foreigner/non-Western foreigners), multimorbidity (yes/no), educational status (low/middle/high), age (continuous in years), sex (male/female), body mass index (BMI, kg/m2, continuous), smoking habits (never/former/current), location of the general practice (urban/rural), year of the general practitioner’s graduation (less/more than 10 years ago), and general practice in a group setting (yes/no).

Each potential confounder will be independently added to the aforementioned linear mixed model, and if it is significantly associated with the outcome (*p* ≤ 0.05), it will be considered a confounder. Both unadjusted and adjusted treatment effects will be reported along with their corresponding 95% confidence intervals and *p*-values. Statistical significance is set at *p* < 0.05. To account for multiple potential confounders, inverse probability of treatment weighting using propensity scores will be used as a sensitivity analysis. The use of hierarchical modeling aims to methodically evaluate the significance of confounders, prioritizing them by their theoretical relevance and observed impact on study outcomes. To ensure analytical robustness, R-squared values will further quantify each covariate’s contribution to the model.

The primary outcome will encompass all measurement times, including data collected at 6, 12, and 18 months. The analytic strategy will use longitudinal data collected at 6, 12, and 18 months, incorporating random effects models to account for the nesting of patients within practices. Model comparison techniques will be used to assess the fit of increasingly complex models to ensure that the analysis captures the impact of the intervention while accounting for provider variability within practices.

### Methods for additional analyses (e.g., subgroup analyses) {20b}

In addition to the secondary objective of assessing the shift in perceived quality of care, employing the PACIC against regular care after 18 months, individually for each chronic condition, further subgroup analyses will be conducted based on age, sex, multimorbidity status, and educational level, as these factors could potentially influence perceived quality of care and the use of the ABCC tool.

Linear mixed models are used for these analyses, with the subgroups added as interaction terms to assess whether the effect of the intervention varied across different subgroups. The subgroup analyses are specified a priori in the study protocol to avoid data dredging.

Adjusted analyses will also be conducted to control for potential confounders. Factors known or hypothesized to be associated with perceived quality of care or the use of the ABCC tool will be considered potential confounders and adjusted for in the analysis, including national background, location of general practice, year of graduation of the general practitioner, and whether the general practice is part of a group.

For adjusted analyses, especially when significant confounders are identified, the Inverse Probability of Treatment Weighting method will be used as described by Hernán et al. [29]. This approach, designed to balance the distribution of observed baseline covariates across treatment groups, mitigates potential bias and is fundamental to estimating the causal effects of interventions.

### Methods in analysis to handle protocol non-adherence and any statistical methods to handle missing data {20c}

The intention-to-treat (ITT) principle will be employed for the analysis, in which all participants, regardless of the degree of their protocol adherence, will be assessed in the group they were originally assigned to [30]. In the case of protocol nonadherence, we will also conduct a per-protocol analysis, analyzing only those participants who strictly follow the treatment initially allocated. This analysis provides insights into the potential effects of the intervention under optimal adherence conditions.

We employ multiple imputations to manage missing data [31]. This technique creates several versions of the dataset, each filling in missing values with plausible data based on the existing data. Each dataset is then analyzed separately, and the results are pooled to provide estimates and confidence intervals that account for the uncertainty around the missing data.

These methods are chosen based on the nature and volume of the missing data, as well as the presumed mechanism for missing data (missing completely at random, missing at random, or missing not at random). The methods employed are described and justified transparently in our study protocol and statistical analysis plan.

### Plans to give access to the full protocol, participant level-data and statistical code {31c}

The full protocol of this study will be available for public access via a registry. Moreover, the participant-level dataset, which is fully anonymized to safeguard participant privacy, will be accessible upon a structured request reviewed by a designated data access committee. This committee will ensure that all requests align with the ethical guidelines and are aimed at legitimate research purposes. In addition, the statistical code used for the data analysis in this study is shared upon reasonable request. Participant consent will encompass these data-sharing provisions, and all data sharing will comply with the relevant data protection laws, privacy regulations, and any additional requirements set forth by funding bodies or publishers.

### Cost-effectiveness analysis

To ensure a comprehensive understanding of the economic impact of the ABCC tool, our cost-effectiveness analysis will employ decision analytic modeling in addition to actual cost and effectiveness data. This approach will allow for a differentiated assessment of the value of the tool from a health care perspective, focusing on direct medical costs and quality-adjusted life-year gains. Detailed methods and results of this analysis will be elaborated in a specific health economic evaluation plan that will be prepared for publication.

## Oversight and monitoring

### Composition of the coordinating center and trial steering committee {5d}

Given the low-risk nature of the intervention and the context of this study, the study oversight structure will be streamlined while ensuring the highest standards of conduct and ethical responsibility [32].

Core oversight is provided by a compact Steering Committee that includes principal investigators, a statistician, and patient representatives. This Committee supervises the overall conduct of the trial, including reviewing progress, ensuring adherence to the study protocol, and making necessary decisions pertaining to the study. Regular meetings will be scheduled on a quarterly basis, with additional meetings arranged as necessary based on the study’s progression.

Day-to-day operations, including participant recruitment, data collection, and implementation of the ABCC tool, will be managed by the coordinating center at the study site. This team will meet more frequently, ideally on a weekly basis, to promptly address operational issues and ensure that the study runs smoothly.

### Composition of the data monitoring committee, its role, and reporting structure {21a}

Data management will be handled by a dedicated team member within the coordination center. They will be responsible for maintaining the integrity and quality of the data collected throughout the trial, including managing the study database, conducting data checks, and handling missing or erroneous data.

### Adverse event reporting and harms {22}

Given the nature of the ABCC tool, which is already in routine clinical use, the risk of adverse events or unintended effects directly attributable to the intervention is anticipated to be low. Nonetheless, a systematic procedure will be implemented for the collection, assessment, and management of potential adverse events or unintended effects. This will include solicited feedback during follow-up consultations and spontaneously reported events.

All incidents, regardless of their perceived relationship with the ABCC tool, will be promptly reported to the coordinating center. The team will then review and classify each event in terms of its severity, relationship with the intervention, and whether it was expected or unexpected.

In the event of any serious adverse event, the steering committee will be notified immediately. Appropriate actions will be taken in accordance with regulatory and ethical guidelines, and these incidents will be reported to the relevant ethics committee and regulatory bodies if necessary.

Data regarding adverse events will be included in the final study report to ensure the transparency and comprehensive evaluation of the safety of the ABCC tool in our study setting.

### Frequency and plans for auditing trial conduct {23}

To ensure the integrity of the study and adherence to the protocol, periodic review of the study will be conducted. This process will be conducted semi-annually and will include a review of data collection and management practices, compliance with ethical regulations, and overall progress of the trial against its objectives and timeline.

While principal investigators will be involved in these reviews, an independent audit will be conducted annually to ensure an unbiased evaluation. The results of these audits will be reported to the trial steering committee and any necessary corrective actions will be implemented promptly.

### Plans for communicating important protocol amendments to relevant parties (e.g., trial participants, ethical committees) {25}

Any proposed modifications to the protocol will first be reviewed and approved by the principal investigators and the trial steering committee. Following this, amendments will be submitted to the appropriate Research Ethics Committee for approval.

Once these approvals are secured, the amendments will be communicated promptly to all participating investigators who will then be responsible for implementing changes at their respective sites. Importantly, trial participants will also be informed of any changes that may impact their participation in the study in a manner that is clear and understandable.

These updates will also be registered in the trial registry in which the study is listed, ensuring that the public and potential future participants have access to the most current information regarding the trial. Significant amendments will be made to all the reports and publications related to this trial.

## Dissemination plans {31a}

The findings of this study will be broadly disseminated to facilitate transparency and contribute to the advancement of health care. The participants will be informed of the study results in a comprehensible manner. For health care professionals, the primary dissemination method will be published in peer-reviewed academic journals. The results will be shared with the relevant medical and scientific conferences, seminars, and professional networks. The general public will be informed of the study results through accessible summaries disseminated via popular media outlets and study websites and potentially through public lectures.

Data from the trial will be made available according to the current best practices for data sharing with respect to participant confidentiality and data protection regulations. Any data sharing arrangement is guided by ethical and legal considerations.

This study has no restrictions on its publication. All publications adhered to recognized ethical guidelines, such as the International Committee of Medical Journal Editors (ICMJE) recommendations for authorship.

## Discussion

The South Tyrolean study will examine the effectiveness and cost-effectiveness of the ABCC tool, which is designed to visualize the burden of disease, promote shared decision-making, and encourage self-management. We hypothesize that the ABCC tool will significantly enhance the perceived quality of care and quality of life and will be cost-effective given its successful implementation in the Netherlands. The strength of our study is its pragmatic approach, involving broad inclusion criteria and a variety of outcomes such as patient experience of care, quality of life, and resource utilization. Our study, like Dutch studies, aims to reflect the effectiveness of the tool in real-world implementation, enhancing external validity.

We aim to minimize confounding factors by considering those known to be associated with interventions and outcomes. Patient allocation will be based on general practice cluster randomization and not on patient characteristics, reducing the risk of selection bias. We acknowledge that many of the study outcomes are subjective and dependent on patients’ perceptions. Additionally, as our study will be conducted only in primary care settings in South Tyrol, we may not be able to extrapolate our findings to other care settings or regions.

Given the intricate design of ABCC studies, operational and practical issues warrant discussion.

First, the implementation of the ABCC tool in general practice is a key operational aspect of this study. Given that the tool is new to the South Tyrolean Health Service, its integration within the clinical workflow of different practices might pose a challenge [33]. It will be crucial to ensure appropriate training for general practitioners and their teams to effectively use the tool and interpret results. Our implementation strategy for the ABCC tool within general practices aims to seamlessly integrate its use during face-to-face consultations. General practitioners will review the results of the ABCC tool with participants immediately, utilizing it as a dynamic component of the patient-provider interaction. This immediate feedback mechanism is designed to enhance shared decision-making and encourage patient self-management in real-time. Furthermore, while the tool is intended to be a valuable resource throughout the patient–provider relationship, its use in each visit will be at the discretion of the healthcare provider, based on the relevance to the patient’s current care needs and consultation focus. This flexible approach aligns with our goal to validate the effectiveness and cost-effectiveness of the ABCC tool in enhancing perceived quality of care and quality of life, as observed in Dutch studies, within the distinct context of the South Tyrolean healthcare system. Successful integration and positive outcomes from this study will inform broader implementation strategies, aiming for a patient-centered and economically beneficial tool across healthcare settings.

Second, the recruitment of a diverse range of general practices, both urban and rural, across different language groups is fundamental to the generalizability of the results [34]. Achieving this diversity might be challenging, given the voluntary nature of participation. Moreover, within each practice, it is important to ensure a balanced representation of patients with different chronic conditions that requires careful management and supervision. To ensure a balanced representation of patients with different chronic conditions within each practice, we will engage with general practitioners to promote the enrollment of a diverse patient cohort. This strategy complements the stratified randomization process, aiming to achieve a comprehensive assessment of the ABCC tool’s impact across the spectrum of chronic conditions addressed in our study.

Third, ensuring participant adherence to the study protocol, particularly with respect to completing the questionnaires at the required time points, is another operational challenge [35]. Strategies to promote participant retention and to manage missing data were incorporated into the study design.

Finally, the study relies on self-reported outcome measures, which could introduce bias [36]. Efforts have been made to select reliable and validated instruments, and the statistical analysis plan includes strategies for adjusting for potential confounding factors. Although these issues pose challenges, they have been carefully considered in the study design and management plan to reinforce the robustness and reliability of the results.

Although the use of tablets and the Curavista App for data collection and management in the ABCC study can provide several benefits, such as real-time data access, streamlined data entry, and improved patient engagement, it also introduces a few potential challenges that need to be addressed [37]. One of the main challenges is the integration of the Curavista App with various software systems employed in various general applications. Ensuring seamless data exchange and synchronization between these systems and their applications is essential for maintaining the data integrity and usability. Moreover, mapping patient data from the app to the corresponding patient charts and dossiers might require custom solutions, considering the diverse formats and structures of electronic health records across practices. Furthermore, the use of tablets for patient-reported outcome measures might present issues related to digital literacy among patients, especially among the elderly or those unfamiliar with such technology [38]. This could affect the quality and completeness of the collected data.

Another concern is data privacy and security, particularly given the use of the Dutch data platform in a study conducted in South Tyrol. The Curavista App is a certified EU data platform, ensuring compliance with different national and regional data protection regulations will be paramount [39, 40]. Clear communication regarding data handling and privacy safeguards is crucial for maintaining participants’ trust and cooperation.

The internationalization of data management for patient care, as in this study, is not yet widespread in Europe but is a growing trend. The increasing use of digital health applications, availability of cloud-based platforms, and need for multinational research collaborations are driving this shift. However, this internationalization also presents challenges related to data privacy, interoperability, and regulatory compliance, which are being actively addressed by ongoing efforts at the EU level to establish a common data space for health [41].

In summary, integrating the Curavista App and conducting a study on tablets may present challenges. Careful planning, robust data management strategies, and adherence to data protection regulations can help mitigate these risks, ultimately enhancing the study’s efficiency and data quality. We plan to conduct context and process evaluations among healthcare providers to gain a deeper understanding of the use of ABCC tools. Additionally, a qualitative study will be conducted among the patients to gauge their satisfaction with the tool.

Our study has significant public health importance if proven effective and cost-effective. The ABCC tool’s potential to improve person-centered care, enhance quality of life, and possibly reduce healthcare costs could greatly contribute to sustainable healthcare in South Tyrol, and possibly extend it to other regions.

In conclusion, despite the challenges and limitations noted, we anticipate that our study will provide valuable insights into the application and effectiveness of the ABCC tool in primary care settings in South Tyrol.

## Trial status

The current version of the study protocol (08.07.2023 version 2) was approved by the Ethics Committee on 09.08.2023 (protocol number 73–2023). As per this report, recruitment for this study has not yet begun. Given these circumstances, it is not possible to provide an approximate date for recruitment completion.

### Supplementary Information

**Additional file 1: Supplementary material.**

## Data Availability

Access to the final anonymized trial dataset will be limited to investigators and researchers with a direct scientific, educational, or public health planning interest. Requesters are required to submit a detailed proposal that demonstrates methodological soundness and a clear justification for the use of the data. In addition, compliance with data protection regulations and ethical guidelines is a priority, and as such, data sharing will be limited to purposes that directly contribute to public health knowledge or practice, subject to approval by the study’s governing bodies.

## Abbreviations

ABC: Assessment of Burden of COPD
ABCC: Assessment of Burden of Chronic Conditions
CCM: Chronic care model
COPD: Chronic obstructive pulmonary disease
EHR: Electronic health record
GP: General practitioner
NHS: National Health Service of Italy
PROM: Patient-reported outcome measure
T2DM: Type 2 diabetes mellitus

## References

[1] Ghebreyesus TA (2018). Acting on NCDs: counting the cost. Lancet.

[2] GBD 2017 Italy Collaborators (2019). Italy’s health performance, 1990–2017: findings from the Global Burden of Disease Study 2017. Lancet Public Health.

[3] Bodenheimer T, Wagner EH, Grumbach K (2002). Improving primary care for patients with chronic illness. JAMA.

[4] Eaton S, Roberts S, Turner B (2015). Delivering person centred care in long term conditions. BMJ.

[5] Longhini J, Canzan F, Mezzalira E, Saiani L, Ambrosi E (2022). Organisational models in primary health care to manage chronic conditions: a scoping review. Health Soc Care Community.

[6] Boudewijns EA, Claessens D, van Schayck OCP, Keijsers LCEM, Salomé PL, in ‘t Veen JCCM (2020). ABC-tool reinvented: development of a disease-specific ‘Assessment of Burden of Chronic Conditions (ABCC)-tool’ for multiple chronic conditions. BMC Fam Pract.

[7] Slok AHM, Twellaar M, Jutbo L, Kotz D, Chavannes NH, Holverda S (2016). ‘To use or not to use’: a qualitative study to evaluate experiences of healthcare providers and patients with the assessment of burden of COPD (ABC) tool. NPJ Prim Care Resp Med.

[8] van Noort EMJ, Claessens D, Moor CC, Berg CALVD, Kasteleyn MJ, In ’t Veen JCCM (2021). Online tool for the assessment of the burden of COVID-19 in patients: development study. JMIR Form Res.

[9] Claessens D, Boudewijns EA, Keijsers LC, Gidding-Slok AH, Winkens B, van Schayck OC. Hitting the bullseye: the psychometric properties of the Assessment of Burden of Chronic Conditions (ABCC)-scale in The Netherlands. Research Square; 2021; Available from: https://assets.researchsquare.com/files/rs-861720/v1/6a26caa8-bbd7-4280-9cb3-8738ad457fe7.pdf?c=1636963158.

[10] O’Donnell A, Kaner E, Shaw C, Haighton C (2018). Primary care physicians’ attitudes to the adoption of electronic medical records: a systematic review and evidence synthesis using the clinical adoption framework. BMC Med Inform Decis Mak.

[11] Loudon K, Treweek S, Sullivan F, Donnan P, Thorpe KE, Zwarenstein M (2015). The PRECIS-2 tool: designing trials that are fit for purpose. BMJ.

[12] Puffer S, Torgerson D, Watson J (2003). Evidence for risk of bias in cluster randomised trials: review of recent trials published in three general medical journals. BMJ.

[13] Slok AHM, in ’t Veen JCCM, Chavannes NH, van der Molen T, Rutten-van Mölken MPMH, Kerstjens HAM (2014). Development of the Assessment of Burden of COPD tool: an integrated tool to measure the burden of COPD. NPJ Prim Care Respir Med..

[14] Nathan RA, Sorkness CA, Kosinski M, Schatz M, Li JT, Marcus P (2004). Development of the asthma control test: a survey for assessing asthma control. J Allergy Clin Immunol.

[15] Test di valutazione dell’asma (ACT) - Il Mio Respiro. Available from: https://www.ilmiorespiro.it/risorse-utili/test-asma-act/. Cited 2023 Jan 27.

[16] Test di valutazione della BPCO - Il Mio Respiro. Available from: https://www.ilmiorespiro.it/risorse-utili/test-cat-bpco/. Cited 2023 Jan 27.

[17] Janssen MF, Pickard AS, Shaw JW (2021). General population normative data for the EQ-5D-3L in the five largest European economies. Eur J Health Econ.

[18] Konerding U, Elkhuizen SG, Faubel R, Forte P, Malmström T, Pavi E (2014). The validity of the EQ-5D-3L items: an investigation with type 2 diabetes patients from six European countries. Health Qual Life Outcomes.

[19] Faller H, Steinbüchel T, Schowalter M, Spertus JA, Störk S, Angermann CE (2005). [The Kansas City Cardiomyopathy Questionnaire (KCCQ) – a new disease-specific quality of life measure for patients with chronic heart failure]. Psychother Psychosom Med Psychol.

[20] Miani D, Rozbowsky P, Gregori D, Pilotto L, Albanese MC, Fresco C (2003). The Kansas City Cardiomyopathy Questionnaire: Italian translation and validation. Ital Heart J.

[21] Hümmert MW, Bütow F, Tkachenko D, Ayzenberg I, Pakeerathan T, Hellwig K (2023). Effects of the COVID-19 pandemic on patients with NMO spectrum disorders and MOG-antibody-associated diseases: COPANMO(G)-study. Neurol Neuroimmunol Neuroinflamm.

[22] Meregaglia M, Malandrini F, Finch AP, Ciani O, Jommi C. EQ-5D-5L population norms for Italy. Appl Health Econ Health Policy. 2022;21:289–303. 10.1007/s40258-022-00772-7 PMC9702834 36434410

[23] Brenk-Franz K, Hibbard JH, Herrmann WJ, Freund T, Szecsenyi J, Djalali S (2013). Validation of the German version of the patient activation measure 13 (PAM13-D) in an international multicentre study of primary care patients. PLoS ONE.

[24] Graffigna G, Barello S, Bonanomi A, Lozza E, Hibbard J (2015). Measuring patient activation in Italy: translation, adaptation and validation of the Italian version of the patient activation measure 13 (PAM13-I). BMC Med Inform Decis Mak.

[25] Dal Negro RW, Distante C, Bonadiman L, Turco P, Iannazzo S (2016). Cost of persistent asthma in Italy. Multidiscip Respir Med.

[26] Slok AHM, Bemelmans TCH, Kotz D, van der Molen T, Kerstjens HAM, in ‘t Veen JCCM (2016). The Assessment of Burden of COPD (ABC) scale: a reliable and valid questionnaire. COPD.

[27] Brueton VC, Tierney J, Stenning S, Harding S, Meredith S, Nazareth I, et al. Strategies to improve retention in randomised trials. Cochrane Database Syst Rev. 2013;(12):MR000032. 10.1002/14651858.MR000032.pub2 PMC4470347 24297482

[28] Kuchinke W, Ohmann C, Yang Q, Salas N, Lauritsen J, Gueyffier F (2010). Heterogeneity prevails: the state of clinical trial data management in Europe-results of a survey of ECRIN centres. Trials.

[29] Hernán MA, Brumback BA, Robins JM (2002). Estimating the causal effect of zidovudine on CD4 count with a marginal structural model for repeated measures. Stat Med.

[30] Hollis S, Campbell F (1999). What is meant by intention to treat analysis? Survey of published randomised controlled trials. BMJ.

[31] Sterne JA, White IR, Carlin JB, Spratt M, Royston P, Kenward MG (2009). Multiple imputation for missing data in epidemiological and clinical research: potential and pitfalls. BMJ.

[32] Boutron I, Guittet L, Estellat C, Moher D, Hróbjartsson A, Ravaud P (2007). Reporting methods of blinding in randomized trials assessing nonpharmacological treatments. PLoS Med.

[33] Ross J, Stevenson F, Lau R, Murray E (2016). Factors that influence the implementation of e-health: a systematic review of systematic reviews (an update). Implement Sci.

[34] Eldridge SM, Ashby D, Feder GS (2005). Informed patient consent to participation in cluster randomized trials: an empirical exploration of trials in primary care. Clin Trials.

[35] Knittle KP, De Gucht V, Hurkmans EJ, Vlieland TPV, Peeters AJ, Ronday HK (2011). Effect of self-efficacy and physical activity goal achievement on arthritis pain and quality of life in patients with rheumatoid arthritis. Arthritis Care Res.

[36] Stone AA, Bachrach CA, Jobe JB, Kurtzman HS, Cain VS. The science of self-report: implications for research and practice. 1st ed. New York: Psychology Press; 1999.

[37] Marcolino MS, Oliveira JAQ, D’Agostino M, Ribeiro AL, Alkmim MBM, Novillo-Ortiz D (2018). The impact of mHealth interventions: systematic review of systematic reviews. JMIR Mhealth Uhealth.

[38] Wildenbos GA, Peute L, Jaspers M (2018). Aging barriers influencing mobile health usability for older adults: a literature based framework (MOLD-US). Int J Med Informatics.

[39] Voigt P, von dem Bussche A, Voigt P, von dem Bussche A (2017). Organisational requirements. The EU General Data Protection Regulation (GDPR): a practical guide.

[40] Voigt P, von dem Bussche A, Voigt P, von dem Bussche A (2017). Rights of data subjects. The EU General Data Protection Regulation (GDPR): a practical guide.

[41] Marcus JS, Martens B, Bucher A, Godlovitch I. The european health data space. Brussels: European Parliament Policy Department for Economic, Scientific and Quality of Life Policies; 2022. Available from: https://www.europarl.europa.eu/RegData/etudes/STUD/2022/740054/IPOL_STU(2022)740054_EN.pdf. Report No.: PE 740.054.

