# Supplementary material for: A cluster-randomized study to evaluate the effectiveness and cost-effectiveness of the Assessment of Burden of Chronic Conditions (ABCC) tool in South Tyrolean primary care for patients with COPD, asthma, type 2 diabetes, and heart failure: the ABCC South Tyrol study
Source: Trials. 2024 Mar 20;25:202. doi: 10.1186/s13063-024-08041-9 (PMC10953192; doi:10.1186/s13063-024-08041-9)
Supplement: Supplementary file 1 — Additional file 1: Supplementary material. [file 13063_2024_8041_MOESM1_ESM.docx]

**Supplementary Materials**

**Table S1.** PRECIS-2 Table for the South Tyrol ABCC Tool Trial

| **PRECIS-2 Domain** | **Description of Trial Component** | **Score (1-5)*** | **Justification** |
| --- | --- | --- | --- |
| Eligibility | Criteria include patients with COPD, asthma, T2D, and heart failure in South Tyrolean primary care | 4 | Broad criteria to reflect a wide patient population similar to a real-world setting. |
| Recruitment | Patients recruited by their general practitioners during routine visits | 5 | Mimics real-world recruitment processes. |
| Setting | Conducted in various general practices across South Tyrol | 5 | Represents a real-world setting typical for primary care in South Tyrol. |
| Organization | Minimal changes to usual care practices, with GPs using the ABCC tool as part of routine care | 4 | Reflects the trial's integration into existing healthcare processes with slight modifications. |
| Flexibility (Delivery) | GPs have discretion in how they use the ABCC tool with patients | 4 | Allows for variation in use, reflecting real-world application. |
| Flexibility (Adherence) | Patients receive care as usual with the option to use additional ABCC tool resources | 4 | Patient adherence is not strictly enforced, mirroring real-life scenarios. |
| Follow-up | Follow-ups at 6, 12, and 18 months, aligned with standard care intervals | 5 | Follow-up intervals are typical for chronic condition monitoring in primary care. |
| Primary Outcome | Patient-reported outcomes on quality of care and health service needs | 5 | Directly relevant to patient-centered care improvements and real-world impacts. |
| Primary Analysis | Intention-to-treat analysis incorporating all randomized patients | 5 | Reflects the analysis method commonly used in pragmatic trials to ensure applicability of results. |

*Score 1-5: Scale from 1 (very explanatory) to 5 (very pragmatic). Abbreviations: COPD, chronic obstructive pulmonary disease; T2D, type 2 diabetes; GPs, general practitioners, ABCC, assessment of burden of chronic condition.
